# Penicillin allergy de-labeling: Adaptation of risk stratification tool for patients and families^☆^

**DOI:** 10.1016/j.waojou.2024.100939

**Published:** 2024-07-23

**Authors:** Simonne L. Horwitz, Ye Shen, Stephanie C. Erdle, Chelsea Elwood, Raymond Mak, John Jacob, Tiffany Wong

**Affiliations:** a Department of Pediatrics, The Hospital for Sick Children, University of Toronto, Toronto, ON, Canada; b BC Children's Hospital Research Institute, Vancouver, BC, Canada; c Division of Allergy, Department of Pediatrics, University of British Columbia, Vancouver, BC, Canada; d Obstetrics, Gynecology and Reproductive Infectious Diseases, University of British Columbia, Vancouver, BC, Canada; e Digital Lab, BC Children's Hospital, Vancouver, BC, Canada; f Department of Pediatrics, Faculty of Medicine, University of British Columbia, Vancouver, BC, Canada

## Abstract

Penicillin allergy is reported in 10% of the population; however, over 90% of patients are deemed non-allergic upon allergist assessment. The goal of this quality improvement project is to validate a patient-driven assessment tool to safely identify patients at low risk of penicillin allergy and de-label them. Pediatric patients and pregnant women referred to the institution's allergy clinics for penicillin allergy assessment were invited to use the patient tool to complete a self-assessment, resulting in the assignment of a risk category. The risk stratification determined using the patient tool was compared against the allergist's assessment.

The patient tool demonstrated agreement with the allergist assessment in 57/84 (67.9%, 95% CI [56.7%,77.4%]) assessments, intra-class correlation (ICC) = 0.618, p < 0.001. In 22/84 (26.2%) assessments, the patient tool determined a higher risk category, primarily due to differences in patients’ perceived timing and description of symptoms. Only 5/84 (6.0%) patients were placed in a lower risk category by the patient tool compared to the allergist assessment. The patient tool demonstrates good validity in determining penicillin allergy risk, offering potential as a method of empowering patients to advocate in their care. Iterative changes to the patient tool will be applied to increase agreement.

To the Editor,

Penicillin allergy is a common drug allergy diagnosis reported in 10% of the population.^1^ However, over 90% of patients with a reported penicillin allergy are found to be non-allergic upon appropriate allergist assessment.^1^, ^2^, ^3^, ^4^ The discrepancy between reported and true penicillin allergy is often due to misclassification of symptoms in patients who receive penicillin-based antibiotics.^5^^,^^6^ For example, children may develop cutaneous symptoms such as urticaria or maculopapular rash; however, these symptoms are often not the result of an IgE-mediated allergy.^5^^,^^6^ Instead, these symptoms may be the result of a bacterial or viral infection or the interaction between penicillin and a pathogen.^5^^,^^6^

Erroneous penicillin allergy labeling is a growing public health problem.^7^ It leads to the use of alternative antibiotics that are generally less effective, more toxic, more costly, and cover a broader range of pathogens.^7^, ^8^, ^9^ The use of broad-spectrum antibiotics is specifically associated with higher healthcare costs, increased rates of *clostridium difficile* infection, and increased rates of antibiotic resistance.^7^, ^8^, ^9^

At our academic hospital specializing in pediatric and women's health, we previously developed and validated a risk assessment structured questionnaire and decision support tool to assess penicillin allergy risk.^10^ The tool stratifies patients into various risk categories based on their clinical history and was found to reliably identify those patients at low risk of true penicillin allergy who can be safely de-labeled.^10^^,^^11^ It has been adapted into an electronic version that is freely accessible on any mobile phone or computer with Internet access. The existing tool is validated to be administered by various healthcare professionals and is specifically designed for non-allergy experts, such as pharmacists, family physicians, and general pediatricians. It is not a patient resource. The goal of this study is to adapt the language of the existing online tool for healthcare professionals into a patient-friendly version and validate it against the gold standard (allergist) assessment, with a focus on assessing construct validity (the ability of the patient tool to measure penicillin allergy risk based on its agreement with the risk stratification found by the allergist) and ensuring its safety. If effective, this can empower lay individuals to conduct their own assessments and gain knowledge about their personal level of allergy risk to better advocate for themselves in their care.

The patient-friendly penicillin allergy de-labeling tool was adapted from the existing allergy de-labeling online assessment tool designed for healthcare providers; it was therefore assumed to have similar content validity. It was developed through iterative consultation with an expert panel involving allergists, researchers, patients, and the original tool developers. The patient tool is an electronic algorithm consisting of several self-assessment questions relating to the timing of the reaction (how many hours after taking a dose of penicillin the reaction occurred, how many days into the course of penicillin the reaction occurred, and how many years ago the reaction occurred) and the relevant symptoms (including cutaneous, respiratory, gastrointestinal, and cardiovascular symptoms as well as symptoms suggestive of an immune complex reaction or severe cutaneous adverse reaction). The tool is intended to enable lay individuals to conduct their own assessments online and provide a summary of risk categorization, basic education, and suggested management to their care provider.

Between April and September 2023, 127 patients, including children 6 months to 17 years old and pregnant adults, referred to the Allergy Clinics at our institution for assessment of penicillin allergy were invited to use the patient tool to complete a self-assessment prior to their scheduled appointment. For all pediatric patients, the tool was completed on their behalf by an adult caregiver. Patients were excluded if they, or their caregiver, did not speak English. The use of the patient tool resulted in the assignment of a risk category: (1) allergic; (2) high risk, possible allergy; (3) low risk, unlikely to be allergic; and (4) not allergic. This is aligned with previous iterations of the tool and gold standard care.^10^ An optional satisfaction survey was included at the end of the patient tool. Eighty-four patients or their caregivers completed the self-assessment prior to their appointments; this was comprised of 41 children (48.8%) and 43 pregnant adults (51.2%). At their appointments the allergist conducted an assessment using the original validated tool. Construct validity (the ability of the patient tool to truly measure penicillin allergy risk) was captured by comparing the new patient-oriented allergy risk assessment tool and the allergist assessment (gold standard) using intra-class correlation (ICC), where ICC = 0 (no agreement) to 1 (excellent agreement). This study is classified as a quality improvement study and we were granted a waiver from the Research Ethics Board. Written informed consent was obtained from all participants.

The primary outcome was the ability of the patient tool to measure penicillin allergy risk compared with the gold standard allergist assessment. The secondary outcomes were to monitor potential safety risks, demonstrated when the patient tool indicates a lower risk category than the gold standard allergist assessment, and to determine patient satisfaction with the tool using a satisfaction survey.

The patient tool was found to have good construct validity^12^ (ICC = 0.618, 95% CI [0.40,0.76], p < 0.001). See Fig. 1. The patient tool and gold standard allergist assessment demonstrated agreement in 57/84 (67.9%, 95% CI [56.7%,77.4%]) assessments. Of these, 44/57 (77.2%) patients were stratified as low risk by both the patient and the allergist and 1/57 (1.5%) patient was stratified as not allergic by both. 33 of these 45 patients have since undergone an oral penicillin challenge; 33/33 (100%) passed, successfully de-labeling the allergy. Of the 57 patients in whom the patient tool and the allergist assessment demonstrated agreement, the remaining 12/57 (21.1%) were stratified as high risk by both the patient and allergist.

**Fig. 1:**
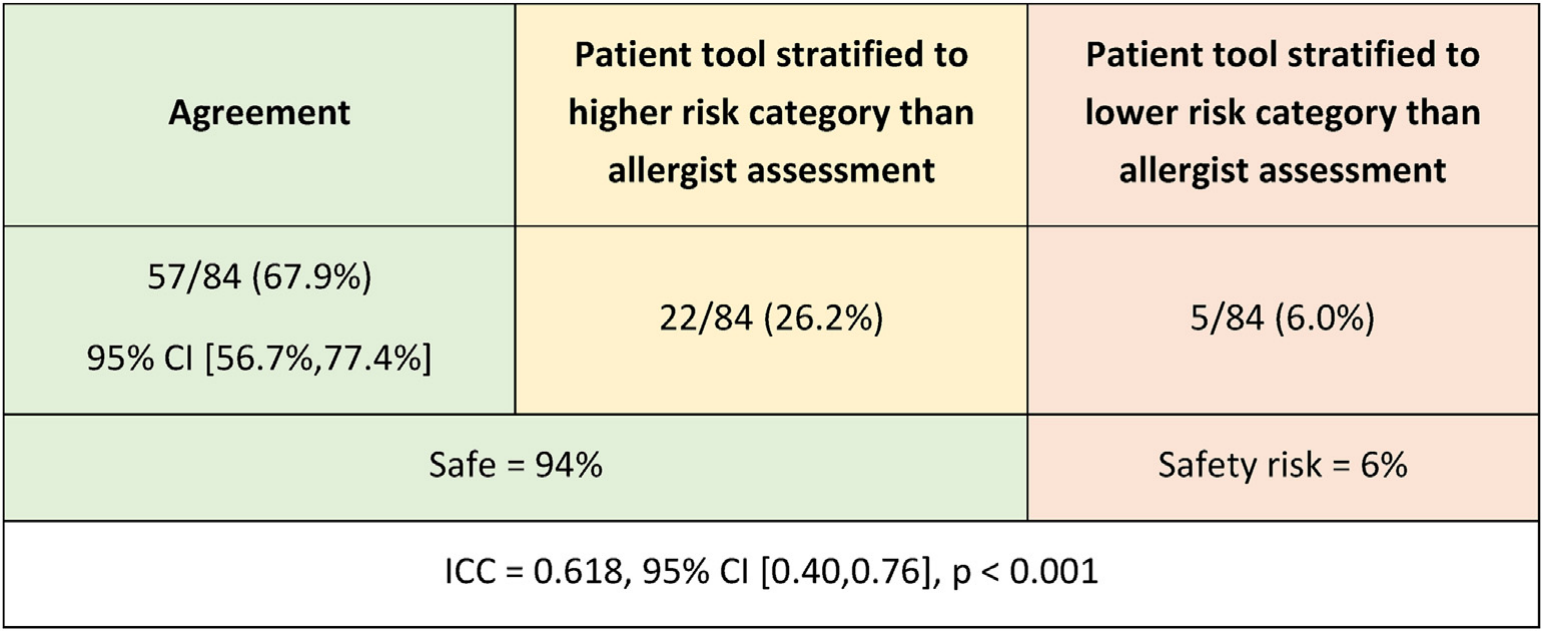
Results of risk stratification by patient tool compared to allergist assessment

In 22/84 (26.2%) assessments, the patient tool determined a higher risk category than the allergist. This was largely due to differences in perceived timing and description of symptoms when completing the patient tool compared to what was reported to the allergist (see Fig. 2). The remaining patients were classified as lower risk by the allergist assessment due to the allergist accounting for either the remote nature of the reaction (more than ten years ago) or a course of penicillin that had been tolerated since the initial reaction, whereas the patient did not report the same on the patient tool. Additionally, a few patients were stratified as higher risk by the self-assessment tool because they indicated that they had been given a penicillin allergy diagnosis by an allergist in the past. See Fig. 2. All the discrepancies differed by only one risk category.

**Fig. 2:**
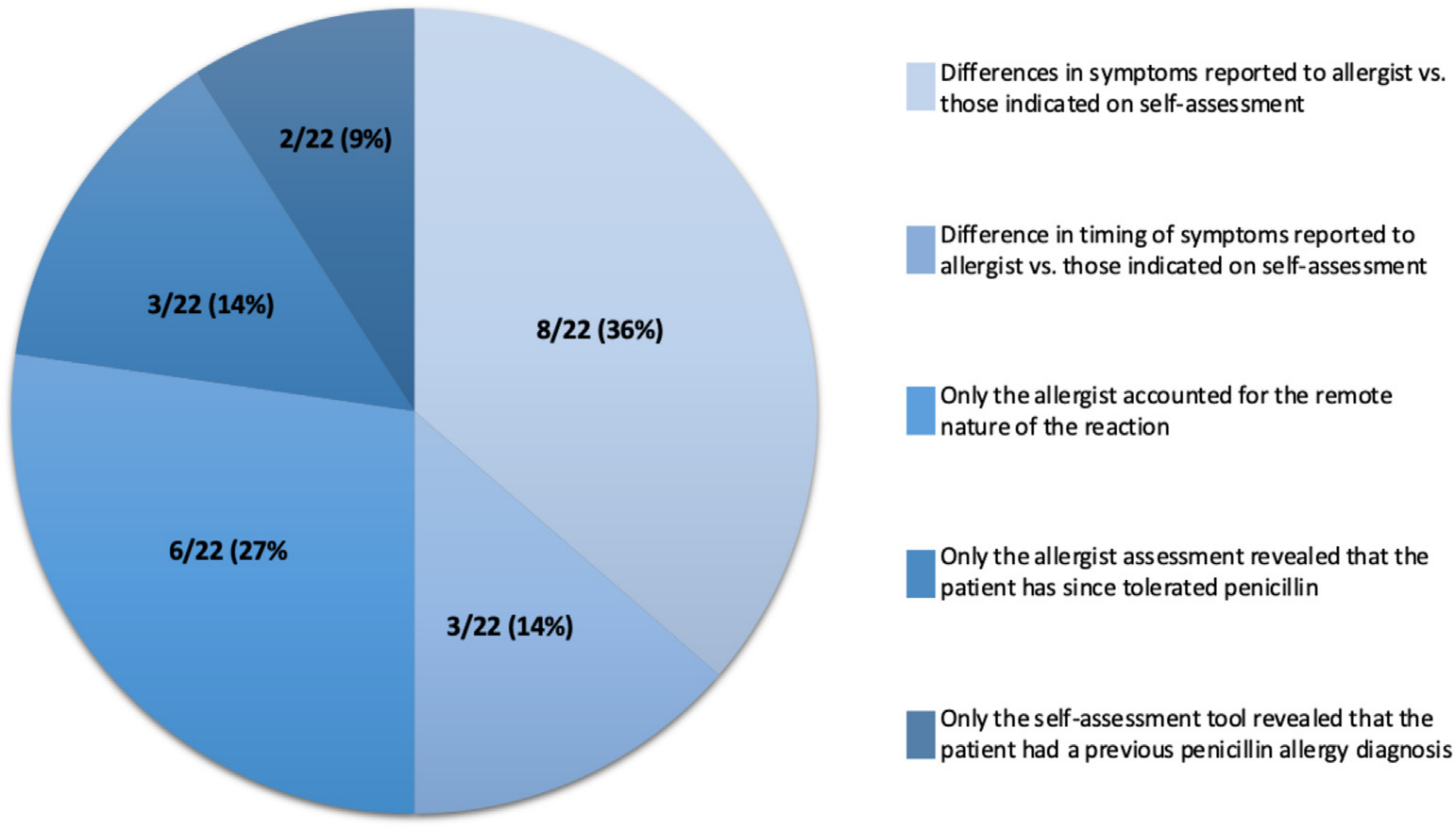
Reasons for disagreement (higher risk stratification by patient tool)

Only 5/84 (6.0%) of patients were stratified to a lower risk category using the patient tool compared to the gold standard allergist assessment, a potential safety risk. Of these 5 patients, one responded to the self-assessment accounting for an initial exposure with mild non-worrisome symptoms, whereas the allergist accounted for a separate second exposure with more concerning symptoms. The other 4 patients misread the first question and incorrectly indicated that they had never taken a penicillin-based antibiotic so were miscategorized as not allergic given no reported history of exposure.

Not all patients responded to the optional satisfaction survey included at the end of the questionnaire. Of those that did respond, 53/62 (85.5%) agreed that the questionnaire was easy to use, 50/61 (82.0%) agreed that the questions were easy to understand, and 44/62 (71.0%) agreed that their family and friends would find the tool useful. Fig. 3 details the results of the satisfaction survey.

**Fig. 3:**
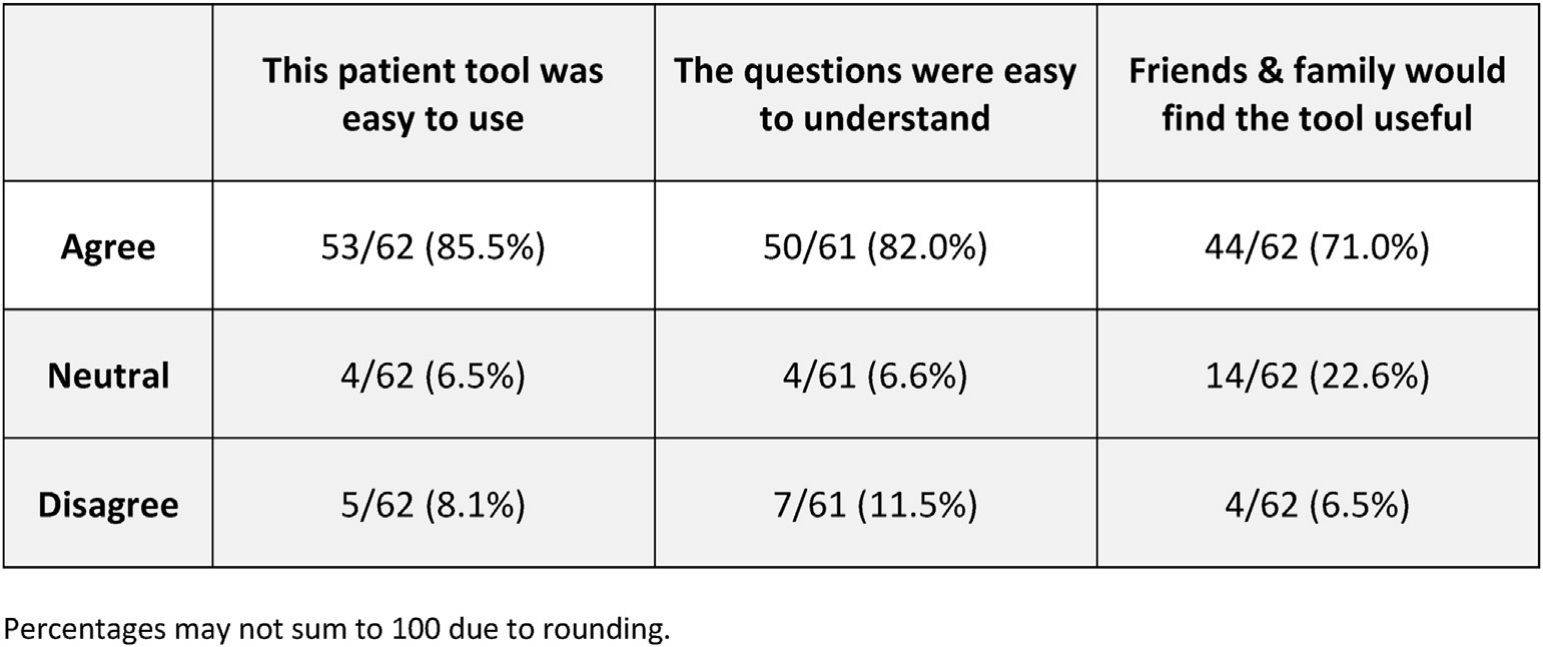
Results of satisfaction survey completed by patient

The patient-friendly version of the original validated de-labeling assessment tool demonstrates good ability to safely determine true risk of penicillin allergy based on its agreement with the gold standard allergist assessment. It therefore offers potential to empower patients to assess their risk of penicillin allergy and advocate in their care, potentially increasing efficiency of healthcare visits, reducing wait times for subspecialist assessments, and preventing personal bias in healthcare providers’ management decisions. To mitigate the safety risk in the small minority of patients and increase overall agreement and accessibility, we will improve readability and flow of the tool based on feedback received in the satisfaction survey. This study provides proof of concept for a patient-driven tool; future work will involve a larger study with adult and pediatric patients from community and hospital settings.

## Abbreviations

ICC: Intra-class correlation.

## Financial support

This work is funded by the Doctors of BC Spreading Quality Improvement program and the Digital Health Innovation Lab at BC Children's Hospital. The funding source had no involvement in the study design; collection, analysis, and interpretation of data; the writing of the report; or the decision to submit the article for publication.

## Availability of data and materials

The datasets used and/or analyzed during the study are available from the corresponding author on reasonable request.

## Author contributions

Simonne L. HORWITZ contributed to data analysis and interpretation and writing of the manuscript.

Ye SHEN contributed to data analysis and interpretation and editing of the manuscript.

Stephanie C. ERDLE contributed to data collection and editing of the manuscript.

Chelsea ELWOOD contributed to data collection.

Raymond MAK contributed to data collection and editing of the manuscript.

John JACOB contributed to study design and editing of the manuscript.

Tiffany WONG contributed to study design, data collection and study execution, editing of the manuscript, and supervision.

## Ethics statement

This study is classified as a quality improvement study and was granted a waiver from the Research Ethics Board.

## Authors’ consent for publication

All authors have read and approved the final manuscript and consent to its publication.

## Declaration of competing interest

Dr. Tiffany Wong is the faculty lead of the Doctors of BC Spreading Quality Improvement program.
