# Corrigendum to “Penicillin allergy de-labeling: Adaptation of risk stratification tool for patients and families” [World Allergy Organ J 17(8) (2024 Jul 23) 100939] doi: 10.1016/j.waojou.2024.100939. eCollection 2024 Aug^☆^

**DOI:** 10.1016/j.waojou.2026.101379

**Published:** 2026-04-27

**Authors:** Simonne L. Horwitz, Ye Shen, Stephanie C. Erdle, Chelsea Elwood, Raymond Mak, John Jacob, Tiffany Wong

**Affiliations:** a Department of Pediatrics, The Hospital for Sick Children, University of Toronto, Toronto, ON, Canada; b BC Children's Hospital Research Institute, Vancouver, BC, Canada; c Division of Allergy, Department of Pediatrics, University of British Columbia, Vancouver, BC, Canada; d Obstetrics, Gynecology and Reproductive Infectious Diseases, University of British Columbia, Vancouver, BC, Canada; e Digital Lab, BC Children's Hospital, Vancouver, BC, Canada; f Department of Pediatrics, Faculty of Medicine, University of British Columbia, Vancouver, BC, Canada

The authors regret to have omitted 2 contributors in the original publication.

We would like to include in the **Acknowledgments** section the work of Chelsea Stunden, MPH, ACRP-CP and Puneet Khosa, BSc, who are affiliated with the Digital Lab, BC Children's Hospital, Vancouver, BC Canada. Ms. Stunden and Ms. Khosa both participated in data curation and project administration. The authors would like to apologise for any inconvenience caused.

Sincerely,

Tiffany Wong MD, FRCPC

Pediatric Allergy | Division of Allergy| Department of Pediatrics, Faculty of Medicine

Faculty Advisor, Spreading Quality Improvement, PHSA Physician Quality Improvement Program

Clinical Associate Professor, University of British Columbia

Clinical Investigator, BC Children's Hospital Research Institute

BC Children's Hospital Rm. 1C31B, 4480 Oak Street | Vancouver BC V6H 3V4 Canada

Tiffany.wong@cw.bc.ca

